# Cardiac contractility modulation: a novel approach for the treatment of heart failure

**DOI:** 10.1007/s10741-016-9571-6

**Published:** 2016-07-09

**Authors:** Freddy Abi-Samra, David Gutterman

**Affiliations:** 1 1516 Jefferson Hwy, New Orleans, LA 70121 USA; 2 MD Medical College of Wisconsin, Milwaukee, WI 53226 USA

**Keywords:** Cardiac contractility modulation, Device, Calcium handling, SERCA2A, Electrical therapy, HFrEF

## Abstract

Heart failure is a major health problem worldwide and, despite effective therapies, is expected to grow by almost 50 % over the next 15 years. Five-year mortality remains high at 50 % over 5 years. Because of the economic burden and large impact on quality of life, substantial effort has focused on treatments with multiple medical (beta-blockers, angiotensin-converting enzyme inhibitors and angiotensin receptor blockers (ARB), aldosterone antagonists, and combination of ARB/neprilysin blockers, ivabradine) and device therapies (ICD, CRT) which have been implemented to reduce disease burden and mortality. However, in the past decade only two new medical therapies and no devices have been approved by the US FDA for the treatment of heart failure. This review highlights the preclinical and clinical literature, and the implantation procedure, related to a relatively new therapeutic device for heart failure; cardiac contractility modulation (CCM). CCM delivers a biphasic high-voltage bipolar signal to the RV septum during the absolute refractory period, eliciting an acute increase in global contractility, and chronically producing a sustained improvement in quality of life, exercise tolerance, and heart failure symptoms. The technology is used commercially in Europe with nearly 3000 patients implanted worldwide. Indications include patients with reduced EF and normal or slightly prolonged QRS duration, thus filling an important therapeutic gap among the 2/3 of patients with heart failure who do not meet criteria for CRT. The mechanism by which CCM provides benefit can be seen at the cellular level where improved calcium handling (phosphorylation of phospholamban, upregulation of SERCA-2A), reversal of the fetal myocyte gene program associated with heart failure, and reverse remodeling are observed. Recent retrospective studies indicate a long-term mortality benefit. A pivotal randomized controlled study is currently being completed in the USA. CCM appears to be an effective, safe technology for the treatment of heart failure with reduced ejection fraction.

## Epidemiology and magnitude of the problem

It is estimated that the prevalence of heart failure among American adults was 5.7 million in 2012 [1], a number expected to climb 46 % by 2030 [2]. This growth in numbers is likely multifactorial. It reflects more effective treatment of acute coronary syndromes resulting in fewer deaths from acute myocardial infarction but more cardiac dysfunction in the remaining survivors. It may also reflect improved treatment with longer survival of patients with dilated cardiomyopathies of various etiologies. Worldwide the prevalence of heart failure is estimated to exceed 25 million [3] but has been difficult to calculate in recent decades due to lack of uniformity in data collection, especially in developing countries. In 2009, heart failure was the most common reason for hospital admission in Germany [4].

**Fig. 1:**
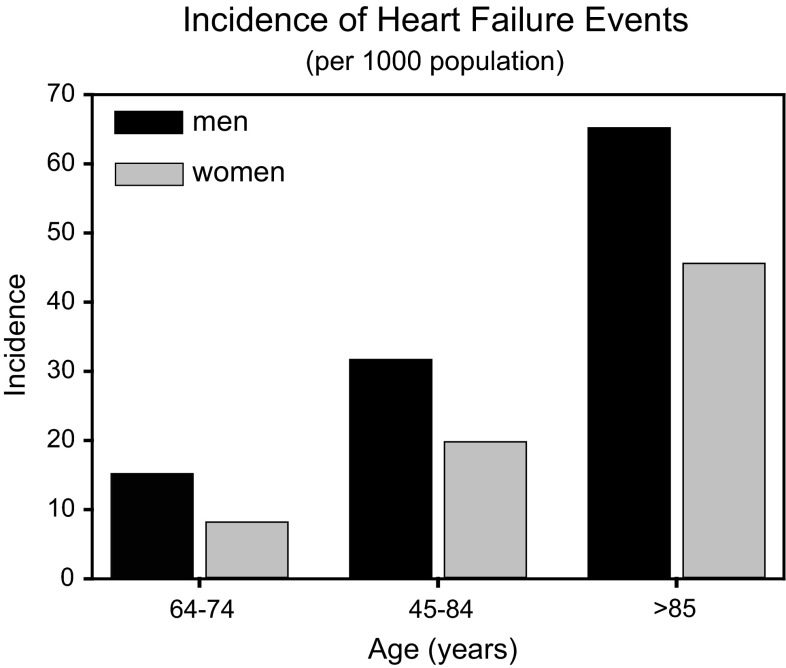
Incidence of heart failure in the USA. An age-dependent increase in new cases of heart failure is observed in older Americans. The incidence is greater across ages in men compared to women. (Adapted from Mozafarian et al. [1].)

**Fig. 2:**
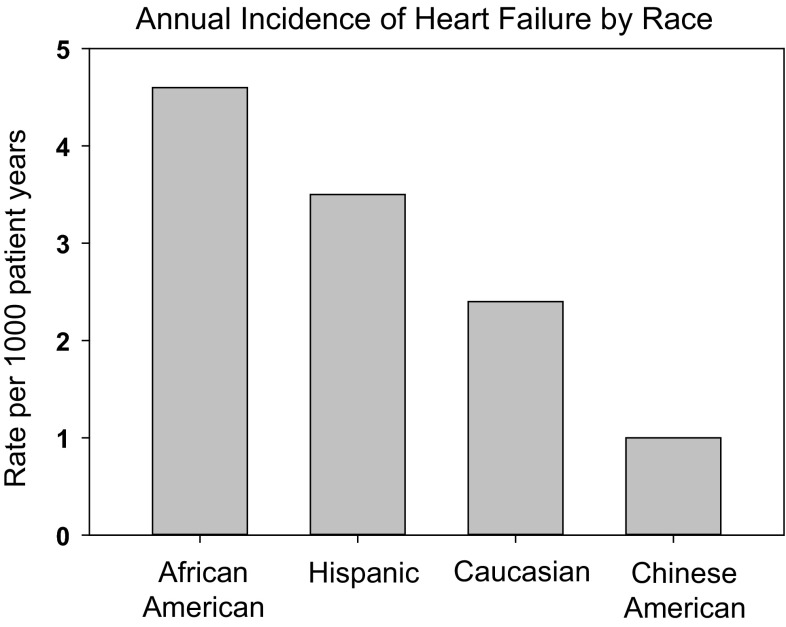
Annual Incidence of heart failure by race in the USA. Heart failure occurs most frequently in African Americans and Hispanics with the lowest incidence in Chinese Americans. (adapted from Mozafarian et al. [1].)

Above the age of 65, heart failure incidence approaches 1 in 100 people. At age 40, the lifetime risk of developing heart failure averages 20 %, but is higher in those with hypertension [1]. Gender and racial disparities are prevalent. Annual rates of heart failure in women are less than in men for all age groups (Fig. 1; adapted from Mozaffarian et al. [1]). The risk of developing heart failure is greater among African Americans than Hispanics, Caucasians, or Chinese Americans (Fig. 2) [1].

In the USA, 1 in 9 death certificates indicates presence of heart failure, with heart failure being the underlying cause of death in more than 58,000 Americans each year [1]. Interestingly, the number of deaths attributed to heart failure was similar in 2011 (284,000) compared to rates in 1995 (287,000) [1]. However, survival has improved over time [5], even though the 5-year mortality rate from heart failure remains high at around 50 % [5, 6]. Much of the improvement in survival relates to the extensive clinical trials yielding several classes of drugs and devices that are effective in reducing symptoms and in many cases improving survival in patients with heart failure.

## Ambulatory medical therapy for heart failure

Over the past few decades, there has been substantial progress in treating heart failure with reduced ejection fraction (HFrEF). The intense scientific and commercial interest in heart failure is the result of its high prevalence. Since the advent of percutaneous interventions for acute coronary syndromes, more patients are surviving their myocardial infarctions but at the expense of developing heart failure. In 2012, heart failure was the #1 reason for hospital admissions in Germany [4] and the second most common reason for hospitalization in the USA among those older than age 65 [7]. As a result, much attention has focused on developing therapies that not only alleviate symptoms, prevent adverse events, and improve quality of life, but also prolong life.

**Fig. 3:**
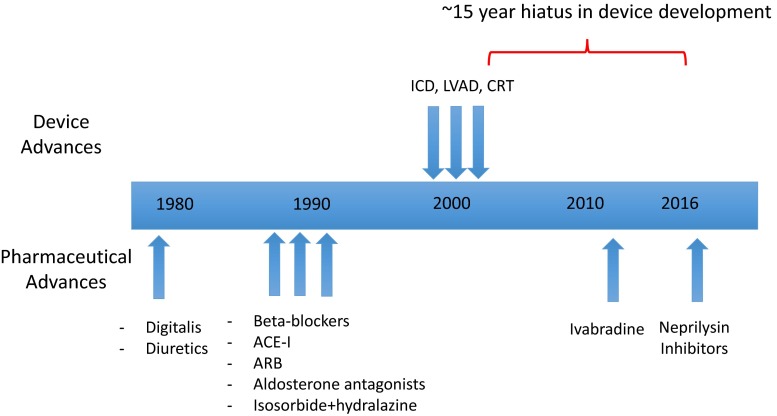
Timeline depicting date of approval of device (*top*) and pharmacological (*bottom*) treatments for HFrEF. There has been a long hiatus in approved device-related therapies. (*Blue arrows*: FDA-approved treatments)

Figure 3 shows the timeline for the introduction of therapies used to treat heart failure. The earliest entries to the list are digitalis and diuretics. Digitalis is rarely used in current practice due to its narrow therapeutic window and lack of data showing a survival benefit. In many patients, risks outweigh benefits, particularly in those with hypokalemia, hypothyroidism, or chronic renal disease. Beta-blockers, angiotensin-converting enzyme inhibitors (ACE-I) or angiotensin receptor blockers (ARBs), and aldosterone antagonists are currently considered optimal medical treatment (OMT) for heart failure due to the mortality benefit afforded by these agents in most patients with HFrEF. They also constitute the primary recommendations in current guidelines on the treatment of heart failure with reduced ejection fraction [8–10]. Another life-prolonging medical regimen for heart failure is the use of hydralazine together with a long-acting oral nitrate [11]. This combined treatment is often used in lieu of ACE-I or ARB in patients at risk of renal dysfunction. However, the combination of hydralazine and isosorbide dinitrate is particularly effective in African American subjects with heart failure and is a first-line therapy in this group [12]. The use of diuretics, particularly loop diuretics, is considered standard practice in HFrEF to treat fluid accumulation peripherally or in the lungs. Although not shown to prolong life, quality of life is improved with diuretic therapy [13], resulting in continued controversy about the risk/benefit with this treatment [14].

The most recent additions to the list of FDA-approved medications for heart failure are ivabradine and a combination of an ARB (valsartan) and a neprilysin inhibitor (sacubitril) [15]. Ivabradine blocks sinus nodal pacing inward cationic “f-currents,” thereby reducing heart rate usually by 15 bpm as its primary mechanism of action [16]. Ivabradine displays a use-dependent effect (i.e., more pronounced inhibition for faster sinus rates). Ivabradine can reduce the composite outcomes of death and rehospitalization in patients with EF <35 % who are in sinus rhythm with HR >70 when given together with guideline-recommended doses of beta-blockers [17]. Ivabradine represents the first new class of drugs approved by the FDA for heart failure, in more than a decade. The combination of valsartan/sacubitril has also been shown to reduce the rate of CV death and heart failure hospitalization and has been added to the list of FDA-approved therapies for CHF.

The numerous randomized controlled trials that have defined the above-mentioned treatments that prolong life in patients with heart failure have resulted in a variety of regularly updated evidence-based guidelines of recommended treatments [8–10, 18]. These treatments not only reduce mortality and/or morbidity, but are also cost-effective [19, 20].

## Device therapy for heart failure

In conjunction with medical treatment, device therapies can also be life-sustaining. In a select population of patients with severely debilitating heart failure for whom life-expectancy is low without heart transplantation, left ventricular assist devices can bridge patients to transplantation. Evolution of device technology with miniaturization and improved durability is creating a niche for LVAD as a destination therapy but only in severe NYHA FC IV patients.

**Fig. 4:**
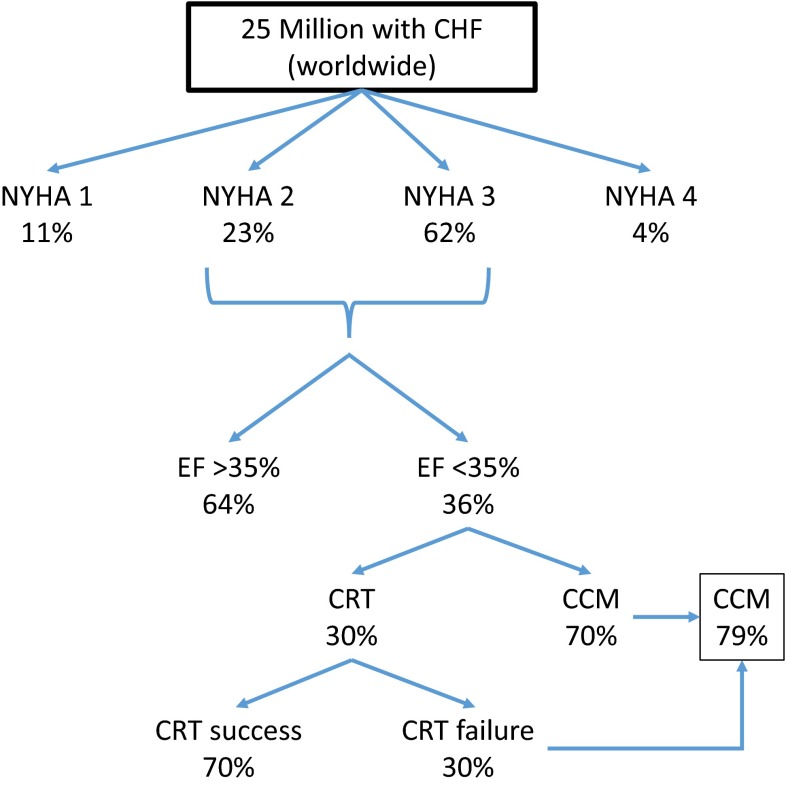
Stratification of patients according to device-related therapeutic options. Of patients with HFrEF with NYHA class II or III, 36 % have an EF <35 %. Of those 30 % with wide QRS durations are candidates for CRT. The remaining patients, including those who fail CRT, constitute 79 % of those with EF < 35 % and are eligible for CCM. Adapted from multiple sources [18, 30, 64]

For subjects with heart failure and ejection fractions less than 35 % despite OMT, implantation of an internal cardiac defibrillator (ICD) is indicated to prolong survival based on several randomized controlled trials [21–23]. However, there is no effect on functional capacity or symptoms with ICD placement. In the subset of symptomatic heart failure patients with low EF and left bundle branch block or otherwise wide QRS duration of 150 ms or more, biventricular pacing (chronic resynchronization therapy; CRT) can improve symptoms and survival [24]. The purported mechanism of action involves coordinating the timing of contractility between ventricles and within the LV. This benefit may be enhanced in patients with less severe reductions in ejection fractions but does not extend to those with normal or modestly prolonged QRS duration, in whom CRT may worsen outcomes [25, 26]. In one registry analysis, less than 10 % of patients admitted to a hospital in heart failure met the electrocardiographic criteria for CRT placement [27]. In a broader analysis of clinical trials of heart failure with EF <35 %, QRS duration of >120 ms is typically seen in only 14–47 % of patients, averaging 30 % [18, 28]. However, in those trials, up to 30 % of patients meeting implantation criteria do not derive a benefit from CRT [29, 30] initially, although long-term follow-up may be required in some patients to observe a benefit. Thus, a large gap exists in our therapeutic device armamentarium for improving function and symptoms in patients with moderate-to-severe systolic dysfunction and normal or mildly prolonged QRS duration. This group represents the majority of patients with symptomatic heart failure patients on OMT and is precisely the population for which CCM is designed to be of benefit (Fig. 4). Among those patients with NYHA class 2 or 3 heart failure with EF ≤35 %, nearly 80 % are estimated to be eligible for CCM therapy (Fig. 4).

### Cardiac contractility modulation

**Fig. 5:**
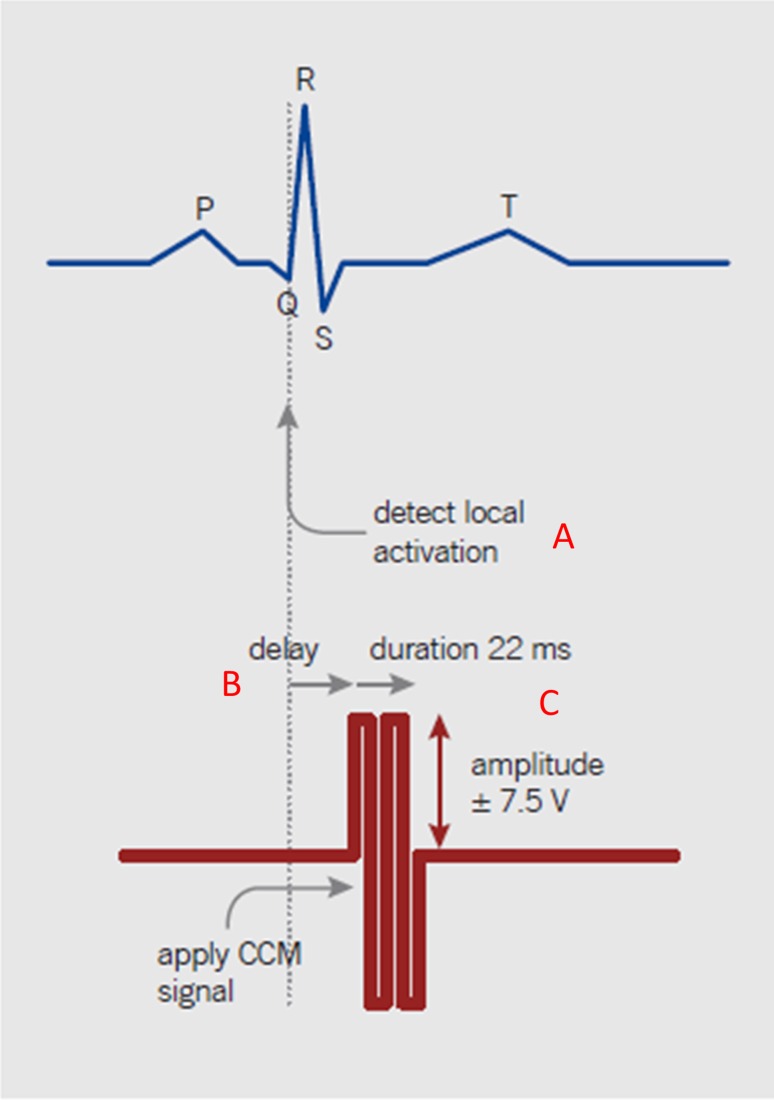
CCM signal triggers from an atrial sensed impulse to augment the next ventricular depolarization which is detected locally from the onset of the QRS (*A*). After a delay of 30 ms (*B*) a biphasic impulse is delivered (7.5 V amplitude, 22 ms duration; *C*), within the absolute refractory period of the ventricle. (reproduced with permission from Kleemann [31])

Cardiac contractility modulation as delivered by Optimizer IV is an established device that is of benefit to patients with symptomatic heart failure on OMT and with normal or mildly prolonged QRS duration, thus providing support for the large complement of heart failure patients who do not have an indication for CRT. CCM delivers a biphasic high-voltage signal (7.5 V/22-ms duration) to the right ventricular septum during the absolute refractory period (Fig. 5) [31]. When administered for 5–12 h/day, this device acutely augments *d*P/*d*t without raising oxygen consumption, thereby improving cardiac efficiency [32, 33]. Chronic improvement in exercise performance and symptoms are reproducibly observed (see clinical studies section below). These improvements are seen in patients with normal or slightly prolonged QRS durations. Similar to CRT, CCM may provide even greater improvement in patients with less severe EF (>25 %) [34].

### Implantation procedure

The implantation of a CCM unit is in many ways similar to that of other cardiac implantable electrical devices (ICD’s and pacemakers). The procedure is best performed under moderate sedation in an OR grade sterile environment with fluoroscopic guidance. For most patients, an ICD is already present in the left prepectoral area, and therefore, most implants would be performed via a contralateral right-sided access. Current devices require the implantation of three standard pacing leads. Two are placed in the RV and one in the RA.

First, the skin and subcutaneous tissues in the (right) deltopectoral groove are infiltrated with local anesthetics. Next, using an extra-thoracic access technique, the axillary and/or subclavian vein(s) are cannulated. The preferred technique favors three separate access sites separated by at least 1 cm to minimize lead-to-lead friction and future binding. Three guide wires are advanced and placed into the IVC. A skin incision is then performed along the three wires, and the incision is carried down to the level of the subcutaneous fascia. A device pocket is then fashioned in the prepectoral area using blunt dissection. The pocket has to be large enough to allow a fully relaxed fit of the device caudal to the inferior margin of the incision. After obtaining full hemostasis, the pocket is irrigated and then packed with dry lap sponges. The leads will then be sequentially placed using three peel-away sheaths introduced over the guide wires. For each lead, mapping for an ideal position is best performed before extending the fixation screw at its tip. This is achieved by connecting the lead pin to a PSA cable and moving each lead from spot to spot until an adequate ventricular or atrial waveform is recorded in the absence of far-field signals and in a position that will provide likely anatomical stability and effective delivery of CCM therapy.

Specifically, the RV lead tips should be placed along the septal wall at least 2 cm apart. Proper placement is best appreciated if multiple oblique views are used. The proposed target zone is the septo-parietal trabeculations, in the inferior portion of the septal RVOT. A road map can be created, and a hand-injection RV angiogram performed via a balloon-tipped open-lumen catheter. The catheter tip is initially placed just under the pulmonic valve, and the injection is performed while simultaneously dragging the balloon catheter from the RVOT into the RV cavity along the septum.

The lead is then positioned using a Mond-type stylet (with a primary J curve and a small posteriorly directed secondary curve). The septal position of the lead is then verified by oblique fluoroscopy views and pacing ECG patterns.

In the 40° LAO view, the lead would be directed rightward and posteriorly toward the spine. The septal position is confirmed in a 30° RAO view.

On ECG, a deep S wave is noted in lead 1 (as opposed to a tall R if pacing from the anterior wall) along with tall R waves in V4–V6 [35] although this finding is debated.

The process is repeated for the second RV lead, the tip of which is positioned slightly more apically or basally (both tips should be in the mid-septal area with at least a 2-cm separation).

Electrical testing of the leads includes the standard testing for pacemaker leads except that excellent sensing function is valued more than pacing capture. Higher impedances are preferred, but this is less important than quality of sensing.

It is important to realize that proper lead tip positioning is essential in a manner similar to LV lead tip positioning. Based on the favorable genomic remodeling that occurs with CCM, a septal tip position (as opposed to a remote site in the RV free wall) would logically afford best signal delivery to the left ventricle.

Once the RV leads are tested and secured into the pocket, the atrial lead is placed with its tip in the RA appendage or the lateral wall. With the PSA cable connected to the lead pin, the lead tip position is varied to identify optimal P wave sensing in an anatomically stable position before extending the distal tip fixation screw.

P wave sensing exclusive of far-field sensing is the essential characteristic sought.

After fixing the atrial suture sleeve, the lead tips are cleaned, dried, and connected to the CCM header. The optimizer box is then placed in the prefashioned subcutaneous pocket with the recharging coil facing anteriorly.

Noninvasive testing is then performed to ensure proper connectivity, sensing, and appropriately timed delivery.

Since most patients also have an implanted ICD, and since the CCM generates a large voltage signal (7.5 V/22-ms duration), testing for device–device interactions is necessary. This testing requires that CCM be delivered while the ICD is set with active tachycardia detection and enhanced sensitivities. Intracardiac electrograms as read by the ICD are then examined for evidence of sensing the CCM signal. It is sensible to check for oversensing prior to CCM device implant by delivering CCM signals with the external simulator. If no oversensing is detected (and therefore no lead repositioning is required), the optimizer unit is then attached to the leads and placed in the pocket. After this step, it is recommended to check again for CCM–ICD interactions.

Implantation often includes testing for appropriate ICD detection of VF waveforms which relies on automatic gain control (AGC) of the sensing circuitry. With AGC, sensitivity is maximized when signals are small (such as during VF) and minimized when R waves are large such as during NSR (to avoid double counting of R waves and/or T waves). Thus, testing of CCM–ICD interactions in VF may yield different results. It is again sensible, when the patient’s hemodynamic status permits, to perform a VF induction/rescue sequence while CCM therapy is active. This is done to ensure that CCM therapy is immediately and properly inhibited during VF lest stimulation signals be sensed by the ICD. This could lead the ICD to misclassify the VF event as sinus rhythm and thus withhold life-saving shock delivery.

Once testing is complete, the CCM pocket is closed in layers (we use 3 layers of absorbable sutures, including a subcuticular layer and we then cover the wound with a layer of medical adhesive ± a silver impregnated dressing). An ice pack is then provided for pain, swelling and bleeding control.

### Mechanism of action of Optimizer therapy

The mechanism of action by which Optimizer improves contractility, exercise tolerance, and symptoms appears multifactorial with acute changes in calcium handling, and chronic improvement in expression and phosphorylation of key calcium regulatory pathways that increase contractility and restore toward normal, the fetal gene expression profile characteristic of heart failure.

#### Acute increase in contractility

Within one beat following initiation of the CCM signal, contractility in isolated rabbit papillary muscle strips increases, but is attenuated as soon as the signal is withdrawn [36]. This acute effect of CCM is also seen in cardiac trabecular muscle of patients with severe heart failure where CCM raises baseline contractility by over 50 % with the onset of stimulation [36]. Within minutes of initiating the CCM signal, myocardial ejection fraction and local contractility typically increase. Using a dog model of heart failure produced by coronary embolization, Morita et al. [37] showed that by 1 h (earliest time point measured), CCM increased EF from 31 ± 1 % at baseline to 41 ± 1 % with sustained further increases for up to 6 h. In humans, a similar acute increase in contractility is observed immediately after implantation, manifest as a rise in EF by 5 %. In fact, several clinical trials have utilized the acute rise in EF to assess lead placement [38–40].

**Fig. 6:**
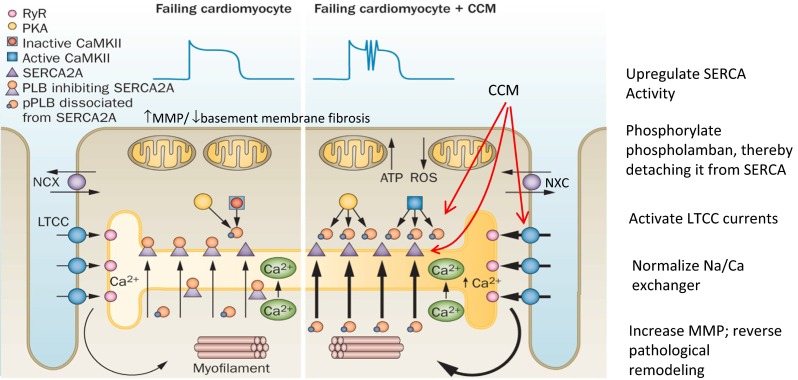
Fundamental mechanism of action by which CCM improves contractility in HFrEF. Key actions of CCM are shown in red and include upregulation of SERCA2A, phosphorylation of phospholamban, activation of L-type calcium channels, restitution of the sodium/calcium exchanger, upregulation of metallomatrix proteins, and reduction in basement membrane fibrosis. (Reproduced with permission from Nature Reviews in Cardiology [41])

Insights into the mechanism of the acute rise in contractility with CCM can be gleaned from studies of calcium handling within the cardiomyocyte. This is reviewed in detail by Lyon et al. [41]. The benefit of CCM involves upregulation of L-type calcium channels and improvement of calcium uptake into the sarcoplasmic reticulum (SR), thereby augmenting (1) extracellular calcium influx during the subsequent membrane depolarization, and (2) calcium-induced calcium release from the SR, respectively. Thus, verapamil, which decreases voltage-dependent calcium entry through the sarcoplasmic membrane, produces a decrease in spontaneous and CCM-augmented contractile effects [42]. Ryanodine which depletes the SR of calcium also decreases CCM-induced calcium release through ryanodine receptor-operated channels, thus inhibiting excitation–contraction coupling and reducing contractility [42] (Fig. 6). Together these data indicate that acute influences of CCM on cardiac contractility occur via multiple mechanisms involving calcium handling in the cardiomyocyte.

#### Chronic effects of CCM in heart failure

**Fig. 7:**
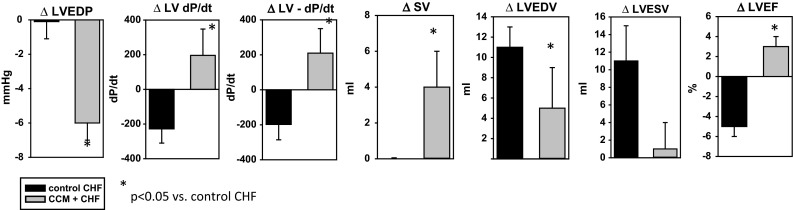
Chronic effect of CCM on cardiac structure and function in a canine model of heart failure. Heart failure was induced by coronary embolism, and dogs were maintained until EF stabilized below 35 % for 2 weeks. CCM (or sham therapy) was started, and animals studied at 3 months. CCM significantly reduced LVEDP and LVEDV, and raised LV dP/dt, stroke volume, and LVEF compared to controls where LV dP/dt and LVEF actually decreased. (Adapted from Morita et al. [43].)

In chronic heart failure models, CCM treatment increases EF, SV, and LV *d*P/*d*t, and retards increases in LVEDV and LVESV, while frankly decreasing LVEDP [43] (Fig. 7). Thus, CCM improves the cardiomyocyte structure and function in heart failure at the cellular and organ levels. To understand how this improvement in cardiac function occurs, it is important to recognize the change in cardiac biochemistry that occurs with heart failure.

**Fig. 8:**
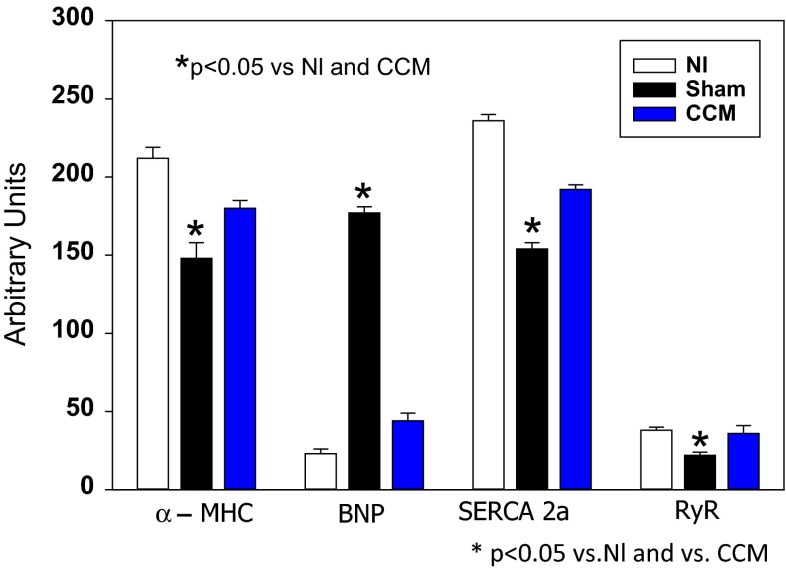
CCM initiates biochemical remodeling and improves the fetal gene program initiated in a canine model of heart failure. After 3 months of CHF, reduced expression (mRNA) of alpha-myosin heavy chains, SERCA2A, and the ryanodine receptor, while upregulating brain natriuretic peptide. CCM normalized these changes. (Adapted from Imau et al. [45].)

Chronic heart failure induces a change in the cardiomyocyte phenotype to that of a more juvenile pattern via reversion to a fetal gene program. Thus, in heart failure, there is increased expression of BNP and the sodium–calcium exchanger, with decreased expression of SERCA2A, alpha-MHC, and phospholamban. Chronic use of CCM in animals with heart failure causes biochemical reverse remodeling of the fetal gene program back toward that of a normal adult (Fig. 8) [44, 45]. In doing so, calcium handling within the cardiomyocyte is improved. Upregulation of SERCA and greater phosphorylation of phospholamban augment SR uptake of calcium resulting in greater release of calcium during the next depolarization, and therefore greater contractility. Other components of calcium handling efficiency in the cardiomyocyte are also improved by CCM including upregulation of the ryanodine receptor and downregulation of the sodium–calcium exchanger [41].

A potential consequence of chronic beneficial cardiac remodeling with CCM is the retrospective clinical observation that QRS duration, which typically prolongs over time in CHF, is maintained constant during CCM (over 2-year follow-up) [46]. This may be important since prolongation of the QRS in CHF is predictive itself of future cardiac death [47]. Future prospective confirmation of this potential benefit of CCM is warranted.

#### Local and remote cardiac effects of CCM

Changes in gene expression regulating calcium homeostasis in cardiomyocytes are seen within 2 h of signal onset, occur locally (in the region of the electrodes) when CCM is active [45], and include an increase in expression of SERCA2A and heightened phosphorylation of phospholamban (PLN) [45]. However, after 3 months of CCM treatment, both local and remote sites demonstrate the same improvement in SERCA2A expression, PLN phosphorylation, and content of MYH6, RyR2, S100A1, sorcin. This represents a general reversion away from the pathological fetal gene expression of heart failure [41, 45].

### Clinical results with device therapy for heart failure

Despite the substantial advances in treating HFrEF, morbidity and mortality remain high with 50 % 5-year survival rates, and 50 % 6-month readmission rates for heart failure exacerbation [48]. For patients with symptomatic heart failure despite optimal medical therapy, device therapy offers promise. CRT is effective for those patients with symptomatic heart failure and prolonged QRS and/or left bundle branch block as described above. However, use of CRT in patients with a narrow QRS duration can actually increase mortality [25]. Approximately 20 % of patients with heart failure have or develop a prolonged QRS duration within the first year of diagnosis [49], and it is estimated that only 30 % of established patients with EF <35 % have a wide QRS (Fig. 4); therefore, only a minority are eligible for CRT. This limits therapeutic options for patients still symptomatic on optimal medical therapy and highlights the need for more treatment options. Interestingly in the past 10 years, only two pharmacological treatments have been approved for use for heart failure in the USA and no new devices have received FDA approval. CCM helps to address this gap in therapeutic options (Fig. 4), has been evaluated in several clinical trials, and is currently approved for use in Europe. This section reviews the clinical data describing efficacy and safety of CCM in patients with HFrEF.

### Optimizer clinical trials

More than 10 clinical trials and ongoing registries spanning 4 generations of the OPTIMIZER device have provided experience with nearly 3000 implanted patients from whom we can document the effects of CCM in HFrEF. These data support a specific clinical profile of patients with HFrEF for whom CCM therapy may provide benefit beyond OMT.

*FIX-HF-3* was the first long-term study of CCM efficacy [40]. Published in 2004, this unblinded observational study enrolled 22 patients from multiple sites across Europe. After 8 weeks of follow-up, there were improvements in quality of life (Minnesota Living with Heart Failure Questionnaire; MLWHFQ), LV ejection fraction, NYHA classification, and 6-min walk test [40].

The results of FIX-HF-3 prompted the conduct of a larger clinical trial, *FIX-HF-4* [39]. This crossover study was patterned after the MUSTIC trial which evaluated cardiac resynchronization [50]. The study design was a comparison between optimal medical therapy (OMT) and OMT + CCM in a crossover, double-blinded, prospective manner with measurements made after 12 weeks of each condition (CCM ON or CCM OFF). Inclusion criteria were NYHA class II–III heart failure with EF <35 %. None of the enrollees had an indication for CRT. Eighty were assigned to group 1 (12 weeks of CCM ON followed by 12 weeks of CCM OFF), and 84 were assigned to group 2 (CCM OFF followed by CCM ON). Both groups were matched in terms of baseline characteristics with average ages of 58.9 ± 9.8 and 59.9 ± 10 in groups 1 and 2, respectively. Over 80 % of both groups were men. Roughly half in each group had an ICD. A small but similar number of subjects dropped out from each group.

**Fig. 9:**
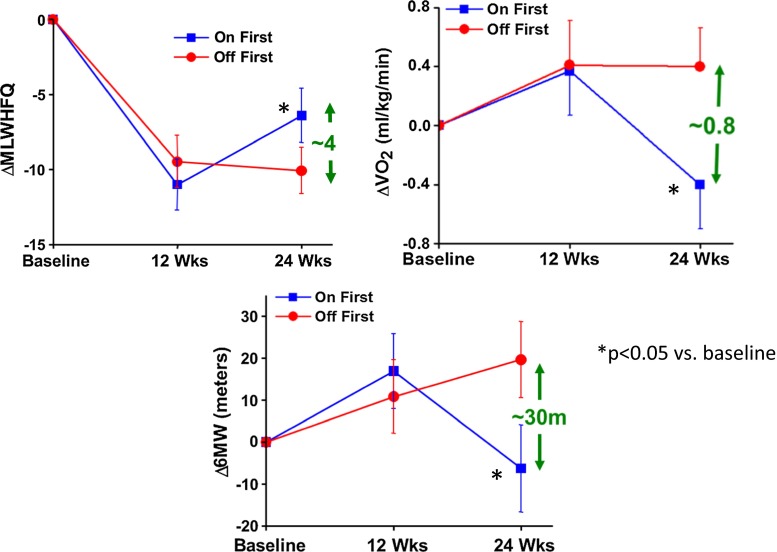
Changes in MLWHFQ (*upper left*), peak VO2 (*upper right*) and 6-min walk test (*bottom*) in patients randomized to CCM ON then CCM OFF (*blue*) or CCM OFF then CCM ON (*red*) from the FIX-HF-4 double-blinded randomized controlled trial. At 12 weeks, a significant improvement was observed in both groups for each parameter. At 24 weeks, only those with CCM ON were able to maintain the improvement. These data suggest a prominent placebo effect at 12 weeks and a sustained clinical beneficial effect at 24 weeks of CCM. (Reproduced with permission from the European Heart Journal [39])

Primary endpoints were a change in peak oxygen consumption (pVO_2_), and secondary endpoints were NYHA, MLWHFQ, and 6-min walk distance [39]. Results are summarized in Fig. 9. Peak oxygen consumption increased similarly in both groups at 12 weeks by ~0.4 mL/kg/min, indicating a significant placebo effect, common among double-blinded device trials. However, at 24 weeks, only those subjects in group 2 (CCM OFF to ON) were able to sustain the improvement in peak vO2, while those in group 1 (CCM ON to OFF) showed a decline (Fig. 9). Quality of life measures responded similarly with MLWHFQ score improving in both CCM and control groups at 12 weeks, but the improvement was maintained only in those patients with CCM ON for the final 12-week period [39]. This suggests that a placebo effect is evident but that it is not sustained for 24 weeks. The magnitude of the benefit from CCM and CRT (MUSTIC trial) were similar, as was the degree of CHF in both groups [50]. However, no placebo effect was observed in the MUSTIC trial, possibly because of single blinding. The safety profile was similar at the end of the first 12 weeks in both groups. FIX-HF-4 demonstrated in blinded fashion an improvement in exercise tolerance and QOL by CCM in patients with symptomatic heart failure despite OMT. This led to a much larger randomized controlled trial designed to obtain needed efficacy and safety data for FDA approval.

*FIX-HF-5* pivotal study is the largest clinical trial of CCM performed to date and the first to extend efficacy and safety observations up to 1 year. The study was conducted within the USA across 50 centers using the Optimizer 3 CCM delivery system. It was a randomized longitudinal comparison of OMT versus OMT + CCM over the course of 1 year with primary endpoint of ventilatory anaerobic threshold (VAT), and secondary endpoints of pVO2, MLWHFQ. Enrollment criteria included EF <35 %, QRS duration <130 ms, NYHA III-IV, and use of stable doses of OMT for at least 3 months. At the time the study was designed, plans were also created to conduct a post hoc analysis of the subset of subjects with EF >25 %. All patients had an implanted ICD, and no enrollees had atrial fibrillation [38]. Although randomized, the study could not be blinded since only those in the CCM arm were implanted. This decision was made due to ethical concerns about conducting an invasive procedure to implant a device which would intentionally remain “off” for at least a year. The unblinded study design created a risk of an unbalanced placebo effect in the CCM arm, and the FDA required use of a primary efficacy endpoint that is less subjective, namely ventilatory anaerobic threshold (VAT). This was the first time that the FDA required VAT as a primary endpoint in any clinical trial for heart failure. Part of the reason that it is not commonly used is that in later stages of heart failure, patients are not able to exercise sufficiently to achieve anaerobic threshold (with lactate production). Nonetheless, 428 patients were randomized, 213 to the control group (OMT), and 215 to the CCM group. CCM was delivered 5 h each day. Baseline characteristics were similar between groups. Most suffered from ischemic cardiomyopathy, and over 90 % were on standard medical regimens for CHF including beta-blockers, ACE-I or ARBs, and a loop diuretic. Efficacy data were analyzed by intention-to-treat analysis.

The safety analysis showed a similar composite adverse event rate for CCM (48.4 %) and OMT (52.1 %). Of these, 30 were serious adverse events related to the optimizer system including lead fracture, dislodgment, infection, and erosion. Over the course of 1 year, SAEs included arrhythmias (in 25 and 29 subjects from OMT and CCM groups, respectively), worsening heart failure (50 and 50 subjects, respectively), localized infection (29 and 27 patients, respectively), and general cardiopulmonary events (46 and 42 patients, respectively). No difference in deaths or individual adverse events was observed. However, when focusing on the period between randomization and study start date (during which time the implantation occurred), as expected there were a few more adverse events in the CCM group (22 events in 13 patients) compared to OMT (9 events in 8 patients).

**Fig. 10:**
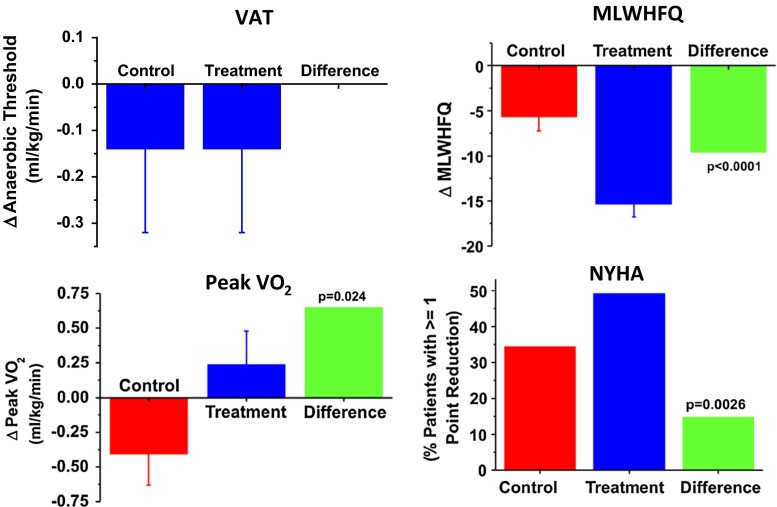
Efficacy results from FIX-HF-5 study of CCM versus optimal medical therapy (OMT) over 1 year. The primary endpoint of ventilatory aerobic threshold was not met although significant improvements in secondary endpoints of peak oxygen consumption, MLWHF questionnaire, and NHYA classification were observed in the CCM group versus OMT. (adapted with permission from Kadish et al. American Heart Journal [38])

**Fig. 11:**
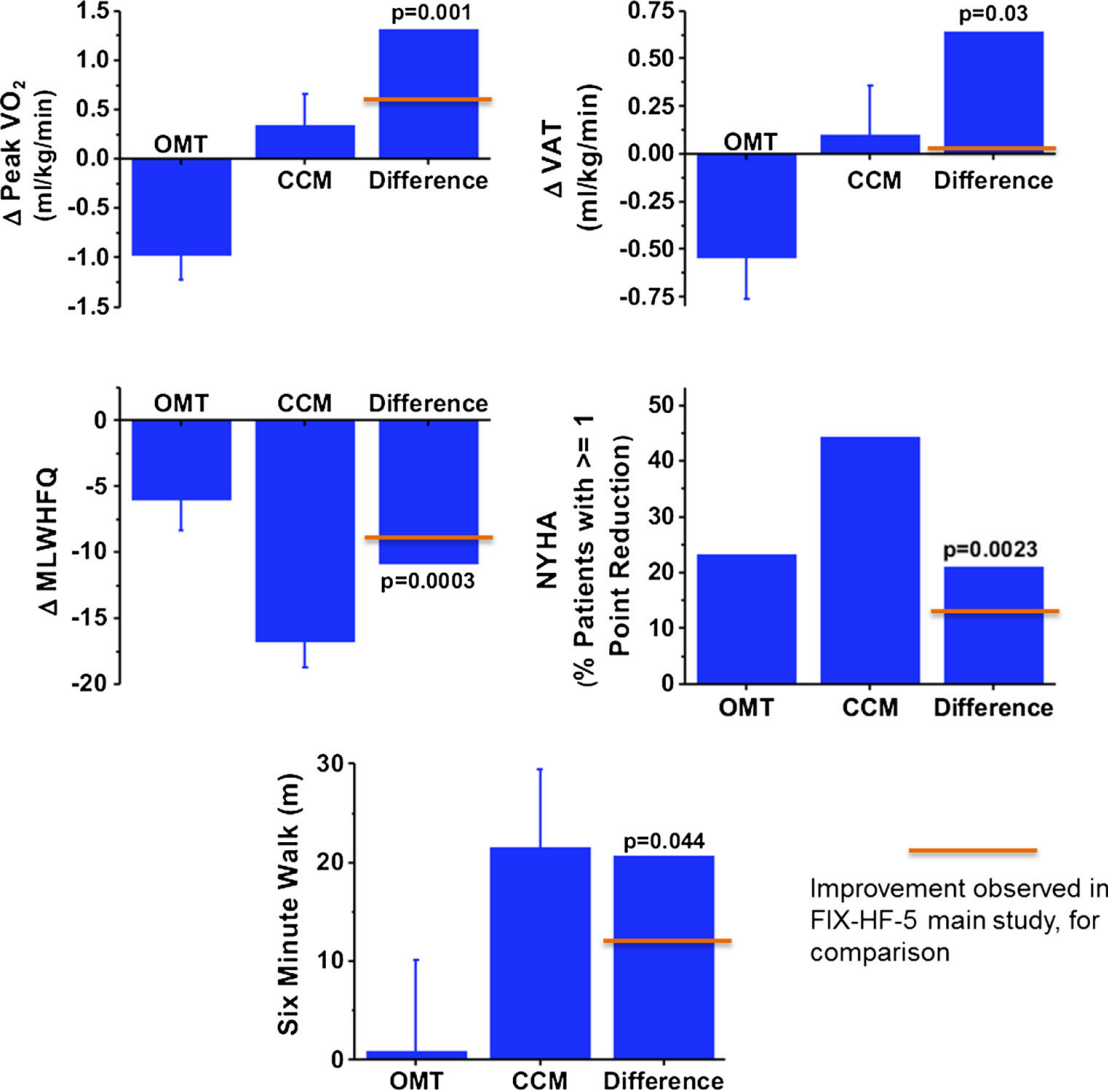
Subgroup analysis of FIX-HF-5 focusing on 150 subjects with LVEF > 25 %. Improvements in VAT, pVO2, NYHA, LVEF, and 6-min walk test were observed. For comparison, orange bars represent amount of improvement seen in the primary study. (adapted with permission from Kadish et al. American Heart Journal [38])

The primary efficacy endpoint, VAT, was not different between OMT and CCM at one year (both decreasing 0.14 ml/kg/min; Fig. 10) [38]. However, secondary endpoints of pVO2, MLWHFQ, and NHYA improved significantly at 1 year in the CCM group. In the planned subanalysis of 150 patients with baseline EF >25 %, a group exclusive of patients with very low EF who are likely sicker and less able to exercise to AT, improvement was observed in VAT, pVO2, NYHA, EF, and 6-min walk test with CCM versus OMT. In each case, the improvement was greater than that seen in the entire cohort (Fig. 11). This provocative hypothesis-generating result suggests that CCM may provide an added benefit in symptomatic patients already on OMT who have moderate reductions in ejection fraction.

Based on the clinical trials already conducted, two meta-analyses have been performed. The earlier analysis by Kwong et al. [51]. pooled 3 studies looking at outcomes of all-cause mortality, all-cause hospitalizations, and adverse effects compared to OMT or OMT+ sham treatment. The authors concluded that CCM, while providing no primary outcome benefit, was not associated with worsening prognosis [51]. The primary efficacy outcomes measures were available in a limited number of subjects, and no effect of CCM was observed. Adverse effects were not different between groups. Interestingly, MLWHFQ, NYHA, pVO2, and 6-min walk test were not analyzed due to lack of data for these outcomes. This meta-analysis was performed on grouped data, and individual patient data were not used.

**Fig. 12:**
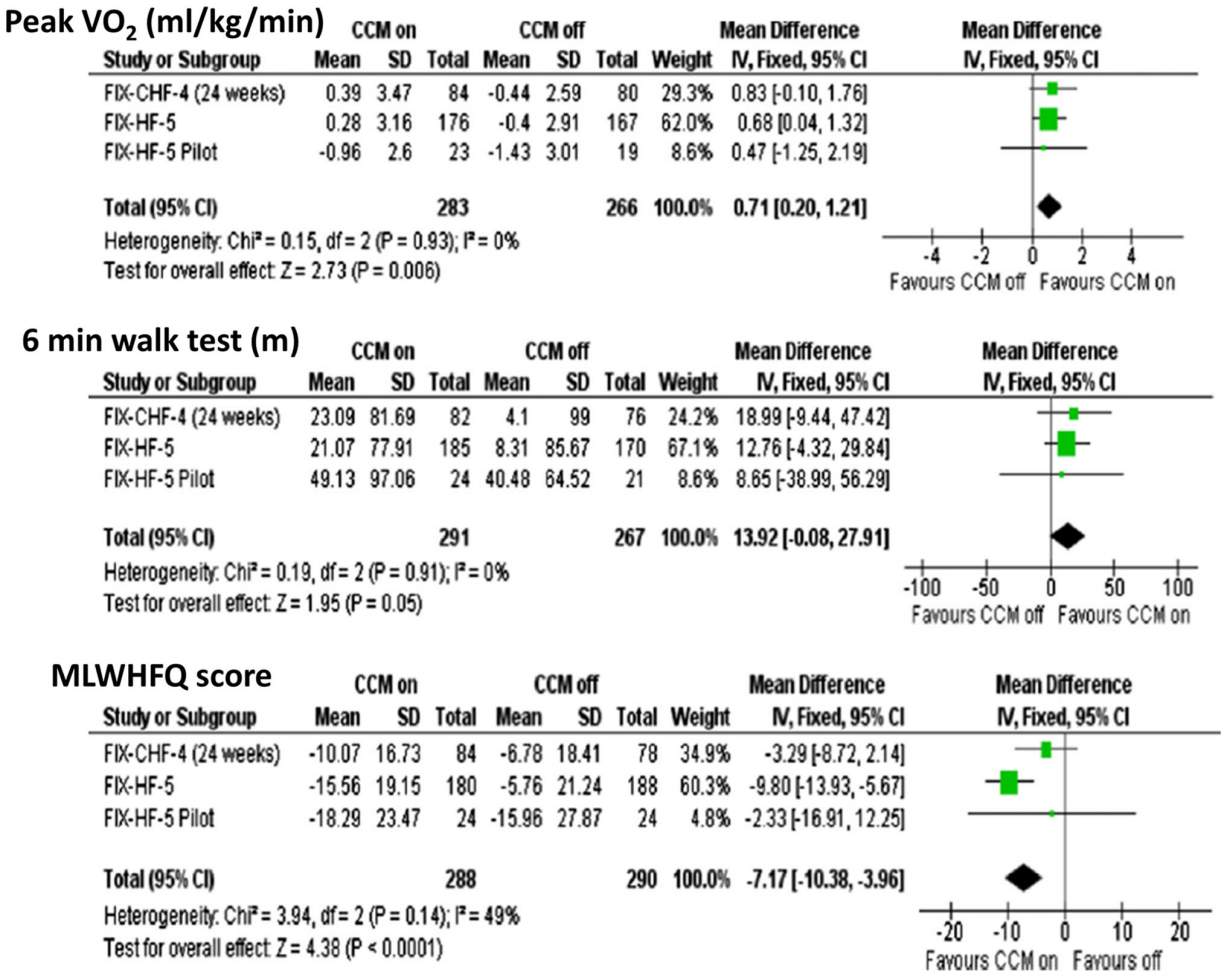
Meta-analysis of major endpoints from key clinical trials of CCM versus optimal medical therapy. Data were analyzed using individual data points. A significant overall benefit was observed favoring CCM for pVO2 and MLWHF questionnaire, with a nearly significant benefit for 6-min walk test. (reproduced with permission from Giallauria et al. [52])

A second and more recent meta-analysis was performed by Giallauria et al. [52] using a similar search strategy to Kwong, resulting in the inclusion of 641 subjects from 3 trials. Even though many of the same data were included in each analysis, Giallauria but not Kwong had access to individual data values, improving the quality of the analysis. In this study, a significant benefit of CCM on outcomes of pVO_2_, 6-min walk test, and MLWHFQ was observed (Fig. 12).

**Fig. 13:**
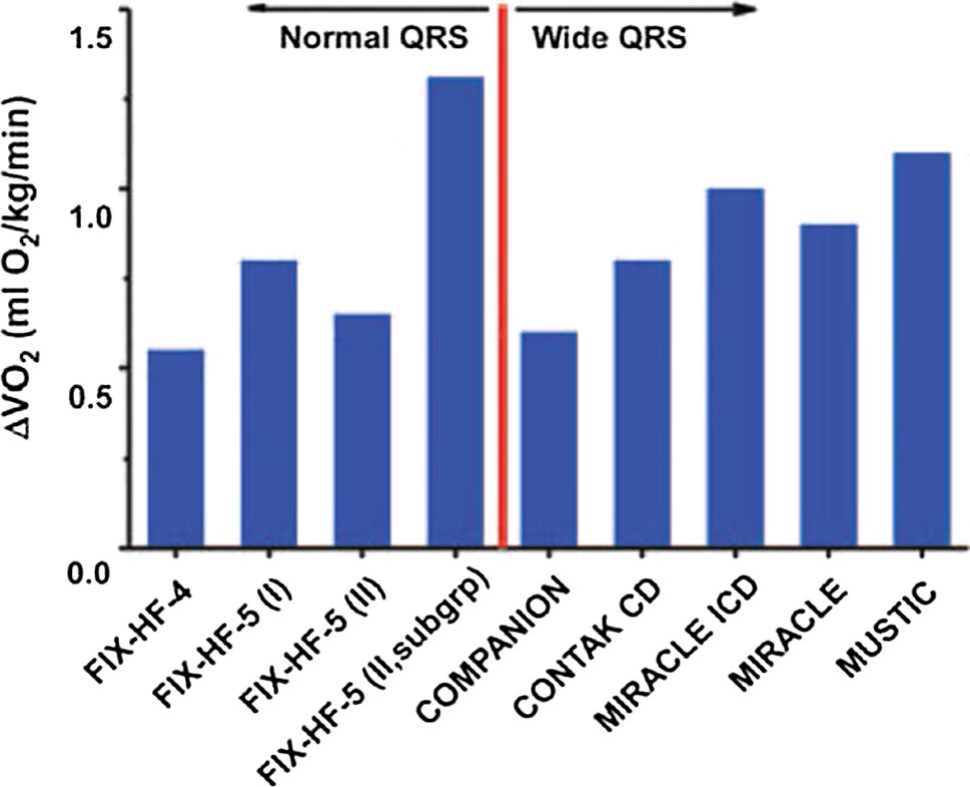
Comparison of the effects of CCM and CRT on peak VO2. Studies to the left of the red line utilized CCM (narrow QRS), while those to the right employed CRT (wide QRS). A similar magnitude of effect was observed in studies from each device. (adapted with permission from European Heart Journal [65])

Collectively, these clinical trials demonstrate a favorable efficacy and safety profile for CCM which parallels that observed in patients with CRT (Fig. 13). However, efficacy with CRT is restricted to subjects with LBBB or very prolonged QRS. Thus, CCM fills a critical gap in our arsenal of therapies to improve symptoms and exercise capacity in patients with heart failure.

### Long-term outcomes

To date, there have been no prospective trials of CCM with mortality as a primary outcome. However, with nearly 3000 implants over a period of more than a decade, there have been several opportunities to observe long-term mortality in patients chronically treated with CCM. Four such published observations are described in this section.

The first of these studies was conducted by Schau et al. [53]. between 2003 and 2010 as a retrospective evaluation of 54 consecutively implanted patients. Patients had moderate-to-severe heart failure with NYHA class III or IV symptoms and average LVEF of 23 ± 6 %. Patients were followed every 3 months for approximately 3 years. Twenty-four patients died during follow-up (18.4 %/year). All-cause mortality was equivalent to that predicted by the Seattle Heart Failure Model [53], a well-validated scoring system that utilizes a diverse group of clinical parameters most of which were available from the CCM patient records.

The second and more recent study by Kuschyk et al. [54] was also conducted at a single site (Mannheim, Germany) with a larger number of patients (81 consecutive patients implanted with a CCM device between 2004 and 2012) who were also followed for 3 years. Patient acuity was similar to that described above with baseline EF = 23 ± 7 % and most presenting with NHYA III or IV symptoms. Kuschyk [54] found long-term improvement in NYHA, MLWHFQ, LVEF, LVEDD, LVESD, and NT-proBNP between baseline and follow-up. Kaplan–Meier survival curves demonstrated a significant improvement in mortality with CCM compared to predicted mortality rates with OMT from the MAGGIC score (14).

A limitation of the MAGGIC scoring system is that it is based on data from a time when ICD use was not routine, and therefore may overestimate mortality when compared to modern-day studies. For this reason, the Kaplan–Meier curve was adjusted to consider every incidence of VT and VF as if it were a mortality event. Even with this strict overcorrection, the event rate in the study population was 13.1 % compared to 18.4 % predicted by MAGGIC at 1 year, and 32.1 versus 40 % at 3 years, respectively.

**Fig. 14:**
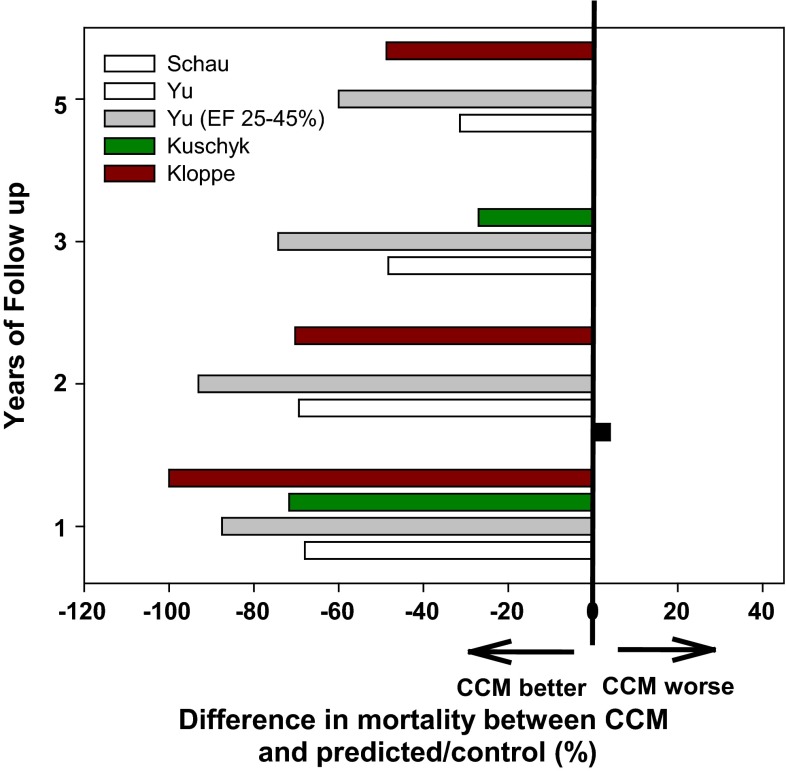
Mortality rates for CCM versus OMT or comparator groups after long-term follow up. Based on the nature of each study, data are presented at 1, 2, 3, and 5 years following device implantation. Results from 5 groups of subjects from 4 clinical trials are displayed. At the longest follow-up time point for each study, mortality rates were the same or lower in the CCM group compared to the medically treated comparator. In one study mortality with CCM was higher in year 2. The benefit was consistent across a broad spectrum of severity of disease (Schau et al. [53] enrolled 54 patients with more severe heart failure—NYHA III–IV, EF = 23 %); Kloppe et al. [56] followed 68 patients with NYHA II–III, mean EF = 26 %); Kuyschyk et al. [54] studied 81 patients NYHA II–IV, EF = 23 %, 14 % of patients had prolonged QRS duration), while Liu et al. [55] tracked 41 subjects with NYHA III and EF = 27 %; ICD use was low in both groups. Comparator groups for each study are as follows: Schau—Seattle Heart Failure Model; Yu, and Yu (EF > 25 %)—subjects on medical therapy alone from the same database matched for age, gender, etiology of heart failure, and duration of heart failure; Kucyck—MAGGIC risk score; Kloppe—Seattle Heart Failure Model

As described in the secondary analysis of FIX-HF-5, CCM appears to confer a greater benefit in patients with EF >25 %. A third and very recently published long-term outcome study addressed this specific subpopulation. Liu et al. [55] examined outcomes in 41 consecutive heart failure patients with EF <40 % in whom a CCM device was implanted. Follow-up extended 6 years and cases were matched 1:1 with controls with respect to age, gender, EF at baseline, medications, follow-up duration and cause of heart failure. The primary endpoint was all-cause mortality. Secondary endpoints included heart failure hospitalizations, cardiovascular deaths, and composite endpoints. CCM and control groups were well balanced with average EF = 28 %. All-cause mortality was lower in the CCM group (Fig. 14). Interestingly, when patients were stratified by EF, no difference was observed in those with EF less than 25 %, but a robust reduction in mortality was observed in the CCM group with EF between 25 and 40 % (Fig. 14). Similar improvements in the entire group and those with higher ejection fractions were seen for cardiovascular deaths and for the composite outcome of heart failure and death. For heart failure hospitalizations, there was no difference between CCM and control groups across the entire cohort or for those with lower ejection fractions. However, a significant reduction was observed with CCM in patients with EF between 25 and 40 %.

The fourth analysis of long-term survival with CCM was recently published by Kloppe et al. [56]. The investigative team evaluated outcomes in 68 consecutive heart failure patients (NYHA II or III) implanted at one of two sites in Germany with CCM devices between 2002 and 2013. Outcomes at 4.5 years were compared with the Seattle Heart Failure Model for matched subjects. Mean LVEF was 26 % ± 6 %; 78 % of patients had an ICD implanted during the study follow-up. The average patient age was 61. Mortality rates at 1, 2, and 5 years were lower with CCM than predicted by SHFM (Fig. 14).

Collectively, these findings suggest a long-term benefit of CCM for cardiovascular and all-cause mortality and are congruent with prior subanalyses suggesting that CCM is particularly beneficial in patients with moderate heart failure. They also support the need for a prospective randomized trial to define a mortality benefit. An ongoing randomized controlled trial will address the effect of CCM in patients with EF between 25 and 40 % on exercise tolerance, quality of life, and symptoms [57].

#### Special applications and evaluations

CCM has also been tested using different stimulus parameters, and in special populations.

##### Duration of stimulus

The choices for daily duration of treatment have been expanded. This is based on a recently published randomized trial of 5 versus 12 h per day of impulse delivery [58]. In this double-blinded pilot study, Kloppe and colleagues studied 19 subjects who were randomized to receive CCM active impulses either for 5 or 12 h/day. Both groups showed similar improvements in MLWHFQ and NYHA, with trends toward improved pVO2 [58]. There were no differences between groups. The maintained benefit from shorter durations of CCM stimulus delivery helps to reduce the battery recharge frequency and may allow some patients with frequent ectopy to still be eligible for implantation.

##### CRT failures

CCM has been tested in the 20 % of patients who fail CRT (nonresponders). In one study, sixteen such patients were treated with CCM and followed for an average of 147 ± 80 days [59]. The patients were older (65 ± 9 years of age) and had more severe heart failure (EF = 27.3 ± 7.4 %) than those recruited for the clinical trials described above. At follow-up, there were significant improvements in NYHA class (3.4–2.8) and ejection fraction (27.3–31.1 %). No electrical interference was noted between CCM and CRT. However, the adverse event rate was high with 3 patients suffering sudden death, 4 developing atrial fibrillation, and two pneumonia. This event rate likely reflects the heightened acuity of these patients who had failed both OMT and CRT therapies.

*Heart failure with preserved ejection fraction (HFpEF)* accounts for up to 50 % of all symptomatic heart failure patients. However, conventional heart failure therapy is ineffective or frankly harmful in this condition, and currently, there is no demonstrated beneficial treatment. With this in mind, a very early and provocative report by Tschope describes the experience with CCM in two patients with symptomatic HFpEF [60]. Endomyocardial biopsies and exercise testing were performed in both cases before and after CCM. Improvements were observed in NHYA classification, 6-min walk testing, EF, and MLWHFQ. There was also an increase in diastolic relaxation and EF reserve by dobutamine challenge.

In diastolic heart failure, underphosphorylation of the cardiac protein titin results in fibrosis and stiffening. CCM therapy upregulated phosphorylated titin, with similar improvement in troponin 3 and myosin light chain 2 [60]. CCM resulted in a reduction in cardiac fibrosis as evidenced by decreased collagen expression by over 20 %. This exploratory study indicates a multi-pronged beneficial effect of CCM in patients with diastolic heart failure.

##### Atrial fibrillation

Atrial fibrillation is a frequent and particularly difficult complication in heart failure, resulting in excessive symptoms due to loss of synchrony in filling an often stiff left ventricle during a typically shortened diastolic period. Although permanent atrial fibrillation prevents the optimizer stimulus due to loss of the atrial sensing, current versions of the device can overcome this limitation, allowing effective impulse delivery in the presence of atrial fibrillation. Early experience with this novel algorithm (linked to the presence of a CRT device impulse) was published by Roger et al. [61]. who described use of CCM in 5 patients who developed permanent atrial fibrillation at or after optimizer implantation. Gating off of the CRT signal, successful CCM therapy was implemented for 12 h/day. After more than a year follow-up, all patients were alive and in each case the clinical condition improved. All 5 showed improvement in NHYA class and MLWHF questionnaire. EF increased or remained unchanged in all subjects. Adverse effects were minimal with one patient developing moderate TR. It was concluded that CCM is a promising treatment for CHF with atrial fibrillation, to improve cardiac function and symptoms. Future adjustments in sensing algorithms should allow for the delivery of CCM without the use of the subterfuge of asynchronous pacing into a fibrillating atrium.

##### Cost-benefit analysis

Evidence-based guidelines increasingly incorporate cost-benefit analysis into crafting recommendations. Heart failure is a prime target for cost analysis since the presence of heart failure may double or triple the cost of a patient’s hospitalization [62]. A recent economic evaluation of CCM was conducted for the UK based on a Markov model using data from prospective CCM trials comparing CCM + OMT with OMT alone [63]. Estimates of life years (LY), quality-adjusted life years (QALY), and treatment costs were made. Clinical variables incorporated into the model included peri-implant complication rates, NYHA class over time, and MLWHFQ from which mortality and hospitalization rates were estimated. Total calculated cost for CCM + OMT was £37,467 versus £16,885 for OMT alone. Life years gained were calculated as 7.96 for CCM + OMT versus 7.0 for OMT alone so that QALY was 5.26 versus 4.0, respectively. This resulted in an incremental cost per QALY of £16,405. These calculations are in line with QALY benefit with CRT and ICD devices and are below the threshold cost used by the UK of between £20,000 and £30,000 per QALY and those used typically in the USA of ~$50,000 [19] for a beneficial therapy. Based on these results, the authors concluded that CCM therapy may be considered cost-effective. However, a more refined analysis should be made with direct mortality and hospitalization data from large prospective RCTs.
